# Fluorine-doped argyrodite sulfide electrolyte enables commercial LiCoO_2_ use for 4.6 V high-voltage all-solid-state batteries

**DOI:** 10.1093/nsr/nwaf217

**Published:** 2025-06-02

**Authors:** Cong Dong, Zhihong Bi, Rui Li, Yuxin Ma, Bin Li, Haodong Shi, Zhizhen Zhang, Zhong-Shuai Wu

**Affiliations:** State Key Laboratory of Catalysis, Dalian Institute of Chemical Physics, Chinese Academy of Sciences, Dalian 116023, China; University of Chinese Academy of Sciences, Beijing 100049, China; China Mobile Energy Technology (Beijing) Co Ltd, Beijing 100080, China; School of Materials, Sun Yat-sen University, Guangzhou 510006, China; State Key Laboratory of Catalysis, Dalian Institute of Chemical Physics, Chinese Academy of Sciences, Dalian 116023, China; University of Chinese Academy of Sciences, Beijing 100049, China; State Key Laboratory of Catalysis, Dalian Institute of Chemical Physics, Chinese Academy of Sciences, Dalian 116023, China; University of Chinese Academy of Sciences, Beijing 100049, China; State Key Laboratory of Catalysis, Dalian Institute of Chemical Physics, Chinese Academy of Sciences, Dalian 116023, China; School of Materials, Sun Yat-sen University, Guangzhou 510006, China; State Key Laboratory of Catalysis, Dalian Institute of Chemical Physics, Chinese Academy of Sciences, Dalian 116023, China; Dalian National Laboratory for Clean Energy, Chinese Academy of Sciences, Dalian 116023, China

**Keywords:** all-solid-state batteries, sulfide solid-state electrolyte, fluorinate, cathode interface, LiCoO_2_

## Abstract

Sulfide solid-state electrolytes (SSEs) are promising candidates for next-generation high-safety all-solid-state lithium batteries (ASSLBs). However, they still face challenges such as low anodic stability limits and poor interfacial compatibility with high-voltage cathode active materials. Here, we present a series of fluorine-doped argyrodite sulfide SSEs, Li_5.5_PS_4.5_Cl_1.5–x_F_x_ (LPSCl_1.5–x_F_x_) (0 < x ≤ 1.5), toward high-voltage LiCoO_2_ (LCO)-based ASSLBs, via the *in situ* formation of a stable fluorine-containing passivating interphase on the cathode active materials surface. Notably, fluorine incorporation significantly raises the practical oxidation limit of LPSCl_1.5_ from 2.4 to 3.5 V for LPSClF_0.5_, while maintaining a high room-temperature ionic conductivity of 3.3 mS cm^–1^. This enhancement is attributed to increased lithium-ion disorder and fluorine's high electronegativity. The ASSLBs, fabricated by directly assembling an LPSClF_0.5_ SSE with an uncoated commercial LCO cathode, demonstrate stable cycling with low polarization voltage at 4.3 V (vs. Li^+^/Li), achieving 92.1% capacity retention after 700 cycles at 0.2 C. Remarkably, even under a 4.6 V high-voltage condition, our battery maintains 96.2% capacity retention over 300 cycles, attributed to the *in situ* formation of a stable fluorine-containing cathode–electrolyte interphase on the LCO surface. When coupled with a lithium metal anode, Li|LPSClF_0.5_|LCO ASSLB achieved stable cycling at 4.6 V and delivered 137 mAh g^–1^ after 100 cycles at 0.5 C. Significantly, the Si|LPSCl_1.5_|LPSClF_0.5_|LCO ASSLB, cycled at an ultra-high mass loading LCO of 203.8 mg cm^–2^, exhibits an exceptional areal capacity of 25.7 mAh cm^–2^, demonstrating immense potential of LPSClF_0.5_ SSE for practical high-energy ASSLBs.

## Teaser

This work reports a fluorine-doped argyrodite sulfide electrolyte, enabling high-performance all-solid-state batteries with either a 4.6 V high-voltage lithium metal anode or an ultra-high LiCoO2 cathode loading of 203.8 mg cm^−2^.

## INTRODUCTION

The rapid development of new energy vehicles, portable electronic devices and large-scale energy storage systems has driven the demand for lithium-ion batteries with high energy density, power density and safety [1–3]. All-solid-state lithium batteries (ASSLBs) have attracted considerable attention for their superior safety profiles, enabled by the replacement of combustible organic electrolytes with inorganic solid-state electrolytes (SSEs) [4–6]. Sulfide-based SSEs have demonstrated particular promise in this context, exhibiting record-high ionic conductivities at ambient conditions (exceeding 32 mS cm⁻^1^) [7], advantageous mass transport characteristics from reduced density [8] and excellent deformability for interfacial compliance [9]. A fundamental limitation stems from their intrinsic narrow electrochemical stability window (1.6–2.4 V vs. Li^+^/Li), which induces detrimental interfacial reactions with conventional cathode active materials (CAM), such as LiCoO_2_ (LCO) and LiNi_1−x−y_Co_x_Mn_y_O_2_, during cycling, creating substantial barriers to practical deployment in high-energy-density battery systems [10–12].

Previous strategies to improve sulfide SSE|CAM interfacial compatibility primarily relied on protective coatings such as LiNbO_3_ [13–15], BaTiO_3_ [16], Li_2_CoTi_3_O_8_ [17] and TiNb_2_O_7_ [18]. These artificial coatings, designed to provide lithium-ion conductivity, electronic insulation and electrochemical stability, physically shield SSEs from oxidation by electrochemically CAM [19]. While theoretically enhancing cyclability under high-voltage operation and extending the anodic limits of SSEs [20], such coatings introduce additional processing complexity, increasing manufacturing time and costs compared to the direct use of bulk SSEs. Moreover, conventional coatings like LiNbO_3_ (electronic conductivity: 10^–11^ S cm^–1^, ionic conductivity: ∼10^–6^ S cm^–1^) impose constraints on cathode design due to their low ionic conductivity [21].

Halide SSEs demonstrate superior compatibility with CAM without requiring protective coatings, showcasing room-temperature ionic conductivities of ∼1 mS cm^–1^ and high theoretical anodic limits (e.g. >4 V vs. Li^+^/Li for chlorides and >6 V for fluorides) [22–25]. However, fluorides suffer from low ion conductivity due to fluorine's strong electronegativity, while chlorides exhibit poor compatibility with lithium metal anodes, necessitating additional sulfide SSEs for interface stabilization [26,27]. This hybrid configuration not only increases manufacturing complexity but also reduces energy density and raises costs due to the use of expensive elements like indium and scandium [28,29]. These challenges underscore the urgent need for developing cost-effective SSEs that combine high ionic conductivity with stable electrode interfaces.

Herein, we propose an innovative SSE design strategy of fluorine-doped argyrodite sulfides, Li_5.5_PS_4.5_Cl_1.5–x_F_x_ (LPSCl_1.5–x_F_x_) (0 < x ≤ 1.5), enabling commercial LCO use for 4.6 V high-voltage ASSLBs. The fluorine incorporation elevates the practical oxidation limit of LPSClF_0.5_ SSE beyond 3.5 V while maintaining high room-temperature ionic conductivity of 3.3 mS cm^–1^. Notably, this strategy effectively mitigates the continuous degradation of SSEs and interfacial side reactions by leveraging the *in situ* formation of a stable fluorine-containing passivating interphase on the surface of CAM. Consequently, LPSClF_0.5_ SSE was directly integrated with uncoated commercial LCO, enabling stable operation of ASSLBs at 4.3 and 4.6 V (vs. Li^+^/Li). Further, coupling LPSClF_0.5_ with micron-sized silicon anodes, the ASSLBs based on ultra-high mass loading LCO of 101.9 and 203.8 mg cm^–2^ exhibit an exceptional areal capacity of 15.0 and 25.7 mAh cm^–2^, respectively. Scanning electron microscopy (SEM) combined with time of flight secondary ion mass spectrometry (ToF-SIMS) revealed a fluorine-rich cathode–electrolyte interphase (CEI) dominated by LiF_2_^–^ and CoF_3_^–^ species. It is experimentally and theoretically revealed that the fluorinated passivation layer formed *in situ* significantly enhances both the anodic stability of LPSClF_0.5_ and the interfacial stability with a high-voltage LCO cathode.

## RESULTS AND DISCUSSION

**Figure 1.**
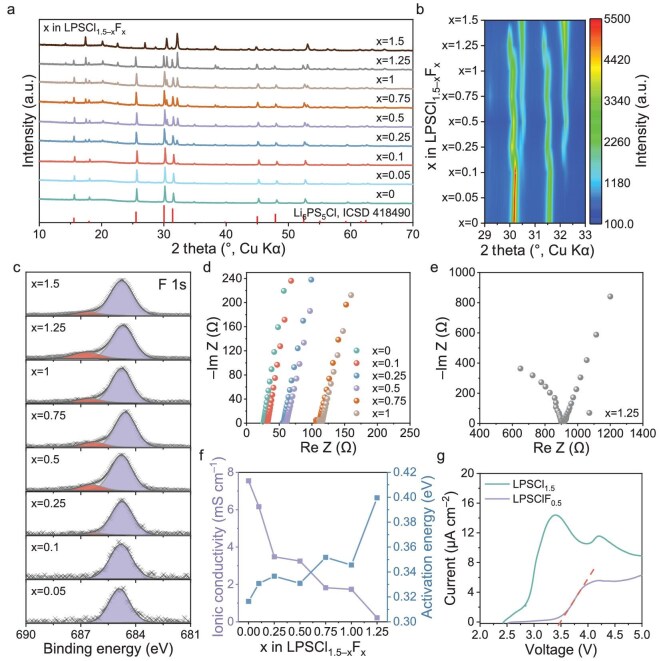
XRD patterns and ionic conductivity analyses for LiPSCl_1.5–x_F_x_ SSEs. (a) XRD patterns of as-prepared LiPSCl_1.5–x_F_x_ electrolytes (x = 0, 0.05, 0.1, 0.25, 0.5, 0.75, 1, 1.25, 1.5) and (b) enlarged diagram of the characteristic peaks. (c) XPS analysis of F 1s spectra of LiPSCl_1.5–x_F_x_ SSEs. (d and e) EIS resolved at characteristic frequencies were acquired from the In|LiPSCl_1.5–x_F_x_|In symmetrical cell configuration at 25°C. (f) Fluorinity dependence of the ionic conductivity and activation energy in LiPSCl_1.5–x_F_x_. (g) LSV analysis of the LPSClF_0.5_ and LPSCl_1.5_ SSEs from the open-circuit voltage to 5.0 V.

The Li_5.5_PS_4.5_Cl_1.5–x_F_x_ (0 < x ≤ 1.5) (denoted as LPSCl_1.5–x_F_x_) was prepared via the ball-milling and solid-state sintering reaction, in which LiF was used to replace some of the LiCl during the fabrication. Figure 1a presents the phase analysis results of annealed LPSCl_1.5–x_F_x_ (x = 0, 0.05, 0.1, 0.25, 0.5, 0.75, 1, 1.25, 1.5). All samples predominantly exhibited an argyrodite Li_6_PS_5_Cl structure (cubic, space group $F\bar{4}3m$) [30], confirming successful synthesis of the target argyrodite phase. The diffuse halo pattern in the low-angle region (15–25°) primarily originates from background interference caused by the air-sealing polyimide film. As x increases (Fig. 1b), the emergence of new diffraction peaks indicates the formation of an F-rich phase, induced by fluorine incorporation. Notably, LPSF_1.5_ does not appear in either the Li-P-S (Fig. S1) or Li-P-F (Fig. S2) ternary phase diagrams, indicating that it likely represents a previously unidentified phase [31]. X-ray photoelectron spectroscopy (XPS) was employed to examine the fluorine chemical environment in fluorinated SEEs LPSCl_1.5–x_F_x_. As shown in Fig. 1c, the characteristic peak at 686.3 eV intensifies with increasing x, indicating the interactions between fluorine and PS_4_^3–^ molecules [31,32]. The stable peak at 684.8 eV confirms Li-F bonding within the LPSCl_1.5–x_F_x_ SSEs [33]. Raman spectroscopy (Fig. S3) further demonstrates that progressive fluorine doping (x = 0 to 1.5) induces both redshift and symmetric broadening of the PS_4_^3–^ characteristic vibration peak (∼427 cm^–1^), revealing the electron-withdrawing effect of fluorine on the PS_4_^3–^ molecular structure [34]. When x ≥ 0.75, the Raman spectra exhibit a band centered at 385 cm^−1^, which is correlated with the P_2_S_6_^4−^ units. The higher content of P_2_S_6_^4−^ units leads to a drastic decrease in ionic conductivity [35].

Electrochemical impedance spectroscopy (EIS) measurements (Fig. 1d and e) display that the Nyquist plots of LPSCl_1.5–x_F_x_ feature nearly diminished semicircular arcs and sharp spikes, representing contributions from grain boundary/bulk resistance and blocking electrode behavior, respectively. The steep linear spike confirms typical ionic conductor characteristics. The incomplete semicircular arcs suggest minimal grain boundary resistance, which is beneficial for sulfide-based solid-state battery assembly. However, due to the inseparable nature of grain boundary and bulk resistances, the total ionic conductivity was calculated using the local minimum resistance at the impedance spectrum's intersection with the real axis. Notably, as x increases, ionic conductivity decreases from 6.2 mS cm^–1^ for LiPSCl_1.4_F_0.1_ to 0.2 mS cm^–1^ for LiPSCl_0.25_F_1.25_, while activation energy rises (Fig. 1f and Fig. S4), indicating that the formation of an F-rich phase hinders lithium-ion transport, consistent with the Raman result. The fluorine-chlorine dual-substituted halide-rich argyrodite sulfide SSE, synthesized via F doping and halide/sulfide disorder modulation based on LPSCl_1.5_, retains high room-temperature ionic conductivity [7,30,36].

Based on a comprehensive evaluation of fluorine content and ionic conductivity, we selected LPSClF_0.5_ (3.3 mS cm^–1^) and LPSCl_1.5_ (7.5 mS cm^–1^) as primary research targets. The electronic conductivity of LPSClF_0.5_ SSE measured by direct current polarization is 6.27 × 10^–10^ S cm^–1^ (Fig. S5), which is lower than that of LPSCl_1.5_ SSE (1.56 × 10^–9^ S cm^–1^). Therefore, fluorine doping endows LPSClF_0.5_ SSE with improved electronic insulation properties. The morphologies of the synthesized LPSClF_0.5_ and LPSCl_1.5_ SSEs were investigated using SEM (Figs S6 and S7). Similar to other reported argyrodite SSEs, both LPSClF_0.5_ and LPSCl_1.5_ exhibited irregular particles with micrometer-scale dimensions [37]. Notably, LPSClF_0.5_ displayed a denser microstructure compared to LPSCl_1.5_. Energy-dispersive spectroscopy (EDS) elemental mapping of selected regions confirmed the homogeneous distribution of P, S, Cl and F across the samples (Figs S6 and S7). The electrochemical stability of SSEs was evaluated using linear sweep voltammetry (LSV) in an asymmetric cell setup, employing a working electrode composed of vapor-grown carbon fiber (VGCF) and SSE composites, alongside a lithium metal counter/reference electrode. The incorporation of VGCF into the working electrode guarantees adequate electrical conductivity, allowing for accurate tracking of reaction potentials. Figure 1g displays the anodic polarization curves (scan rate of 0.1 mV s^–1^) for VGCF/LPSClF_0.5_ and VGCF/LPSCl_1.5_ systems. The LPSClF_0.5_ cell exhibits an anodic potential of 3.5 V, beyond which Faradaic current is observed due to electrode oxidation. The sustained minimal current likely originates from non-Faradaic processes associated with a passivating interface formed at the electrode surface. In contrast, the LPSCl_1.5_ system shows a lower anodic onset potential of 2.4 V, followed by significant Faradaic current, indicative of inferior electrochemical stability.

**Figure 2.**
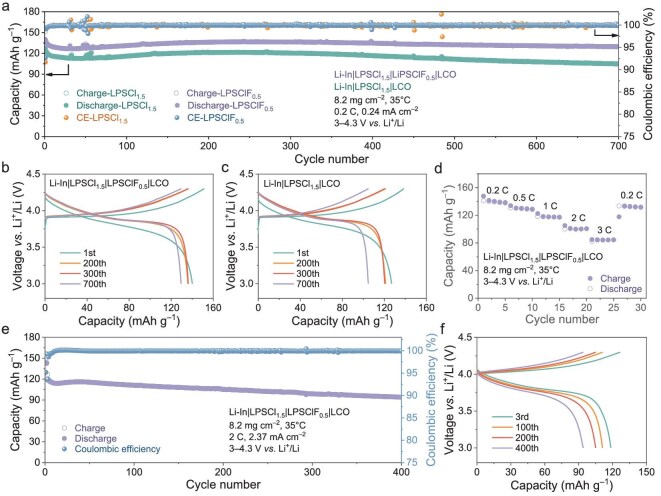
Electrochemical performance of Li-In|LPSCl_1.5_|LPSClF_0.5_|LCO ASSLBs, operated within 3.0–4.3 V vs. Li^+^/Li at 35°C. (a) Capacity and Coulombic efficiency for 700 cycles of Li-In|LPSCl_1.5_|LPSClF_0.5_|LCO and Li-In|LPSCl_1.5_|LCO ASSLBs obtained at 0.2 C (0.24 mA cm^–2^). (b and c) GCD profiles of Li-In|LPSCl_1.5_|LPSClF_0.5_|LCO (b) and Li-In|LPSCl_1.5_|LCO (c). (d) Rate capability of Li-In|LPSCl_1.5_|LPSClF_0.5_|LCO. (e) Capacity and Coulombic efficiency of Li-In|LPSCl_1.5_|LPSClF_0.5_|LCO measured at 2 C (2.37 mA cm^–2^) at 35°C, and (f) corresponding GCD profiles cycled at the 3rd, 100th, 200th and 400th times.

To compare the electrochemical performance of LPSClF_0.5_ with LPSCl_1.5_, we constructed ASSLBs using uncoated commercial LCO cathodes and Li-In (or Li-Si) anodes. In the battery design, a thin layer of LPSCl_1.5_ SSE was added on top of the anode due to its high ionic conductivity, while either LPSClF_0.5_ or LPSCl_1.5_ served as the separator layer between the cathode composite and LPSCl_1.5_ SSE. The cathode composite consisted of 80 wt% LCO and 20 wt% of the corresponding SSE (LPSClF_0.5_ or LPSCl_1.5_). Figure 2a–f presents the long-term cyclability and rate capability of Li-In|SSE|LCO batteries using LPSClF_0.5_ and LPSCl_1.5_ at 35°C. The LPSClF_0.5_ system demonstrated superior electrochemical performance to LPSCl_1.5_. The LPSClF_0.5_ system exhibited higher initial Coulombic efficiency of 92.7% (91.7%) and discharge capacity of 140 mAh g^–1^ (127 mAh g^–1^) than LPSCl_1.5_, along with a larger capacity retention of 92.1% (82.7%) after 700 cycles at 0.2 C (Fig. 2a). Galvanostatic charge–discharge (GCD) profiles of the LPSClF_0.5_ battery (Fig. 2b) displayed low polarization voltage and high cycling overlap after 200 cycles, indicating excellent interfacial compatibility between LPSClF_0.5_ and uncoated LCO. In contrast, the LPSCl_1.5_ battery presented rapid capacity decay after 200 cycles (Fig. 2c), attributed to severe parasitic side reactions at the LCO|LPSCl_1.5_ interface, including electrolyte oxidation and impedance accumulation. The ASSLBs based on LPSClF_0.5_ also demonstrated excellent rate capability, delivering reversible capacities of 141, 130, 118, 100 and 82 mAh g^–1^ at 0.2, 0.5, 1, 2 and 3 C, respectively (Fig. 2d). Furthermore, at a high rate of 2 C, the ASSLB maintained a capacity of approximately 94 mAh g^–1^ after 400 cycles, achieving a capacity retention of 79.3% (Fig. 2e and f), highlighting its exceptional high-rate durability.

**Figure 3.**
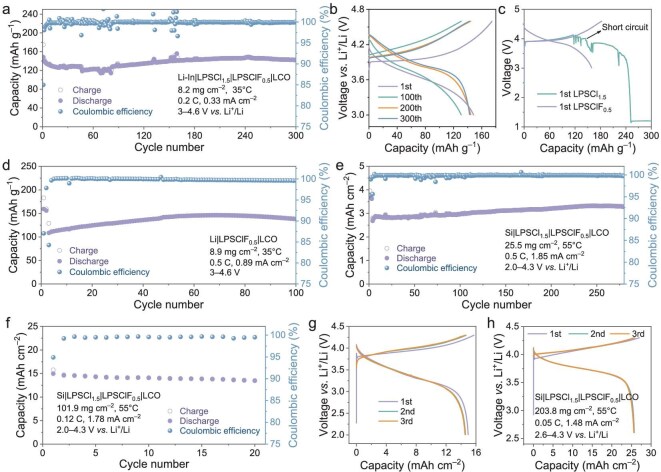
Electrochemical performance of ASSLBs under 4.6 V high voltage vs. Li^+^/Li and high mass loading conditions. (a) Long-term cycling of high-voltage Li-In|LPSCl_1.5_|LPSClF_0.5_|LCO ASSLBs cycled between 3.0 and 4.6 V vs. Li^+^/Li and (b) the corresponding GCD profiles at different cycles. (c) The first GCD profiles of Li|LPSClF_0.5_|LCO and Li|LPSCl_1.5_|LPSClF_0.5_|LCO ASSLBs. (d) Cycling stability of Li|LPSClF_0.5_|LCO ASSLBs at 0.5 C at 4.6 V. (e and f) Cycling stability of Si|LPSCl_1.5_|LPSClF_0.5_|LCO ASSLBs based on high mass loading LCO of 25.5 mg cm^–2^ (2.8 mAh cm^–2^) under 1.85 mA cm^–2^ (e) and 101.9 mg cm^–2^ (15.0 mAh cm^–2^) under 1.78 mA cm^–2^ (f) at 55°C, and (g) the corresponding GCD profiles at the first, second and third cycles. (h) The GCD profiles of Si|LPSCl_1.5_|LPSClF_0.5_|LCO ASSLBs based on ultra-high mass loading LCO of 203.8 mg cm^–2^ (25.7 mAh cm^–2^) under 1.48 mA cm^–2^ at 55°C.

While the aforementioned ASSLBs demonstrate excellent performance at a cutoff voltage of 4.3 V (vs. Li^+^/Li), elevating the cutoff voltage is essential to further uncoil the high stability of SSEs and cathode. Figure 3a illustrates the cycling performance of Li-In|LPSCl_1.5_|LPSClF_0.5_|LCO ASSLBs at an elevated cutoff voltage of 4.6 V. As cycling progressed, the discharge capacity initially decreased, followed by an upward trend before stabilizing, ultimately retaining 142 mAh g^–1^ after 300 cycles, with a capacity retention of 96.2%. GCD profiles (Fig. 3b) further revealed a gradual reduction in polarization voltage and an increase in the discharge median voltage from 3.6 to 3.9 V (vs. Li^+^/Li) (Fig. S8), manifesting the formation of a stable CEI layer at the LPSClF_0.5_|LCO interface.

The incorporation of fluorine significantly enhances the lithium metal stability of the LPSCl_1.5–x_F_x_ SSEs. Consequently, three types of SSEs (LPSCl_1.5_, LPSCl_1.25_F_0.25_ and LPSClF_0.5_) were used to assemble ASSLBs based on lithium metal anode. Notably, the Li|LPSCl_1.5_|LPSClF_0.5_|LCO ASSLB exhibited short-circuit behavior during the first cycle (Fig. 3c). In contrast, the Li|LPSClF_0.5_|LCO ASSLB maintained comparable stability under a 4.6 V high-voltage condition, achieving 137 mAh g^–1^ after 100 cycles at 0.5 C with a capacity evolution pattern similar to Li-In|LPSCl_1.5_|LPSClF_0.5_|LCO ASSLB (Fig. 3d). Interestingly, the Li|LPSCl_1.25_F_0.25_|LPSClF_0.5_|LCO ASSLB employing the low-F-content SSE as the separator initially showed soft-shorts behavior followed by self-repair functionality during cycling (Fig. S9), further underscoring the critical role of fluorine in stabilizing the SSE|anode interface [31]. This observation aligns with research trends emphasizing fluorine's ability to form stable F-rich interfacial layers, which suppress detrimental side reactions and lithium dendrite growth [38,39]. For instance, studies on Li_6_PS_5_Cl-MgF_2_ electrolytes revealed that F-induced LiF layers act as electron insulators, blocking interfacial redox reactions [40].

Although ASSLBs with conventional cathode loadings (8–9 mg cm^–2^) exhibit superior performance, increasing the mass loading to achieve areal capacities (>3 mAh cm^–2^) comparable to commercial liquid lithium-ion batteries remains imperative. Figure S10 presents the cycling results of ASSLBs based on a high mass loading LCO of 25.5 mg cm^–2^ (3.6 mAh cm^–2^), cycled at 55°C under 0.2 C (0.58 mA cm^–2^). The Li-In|LPSCl_1.5_|LPSClF_0.5_|LCO batteries demonstrated enhanced cyclability compared to Li-In|LPSCl_1.5_|LCO, retaining >2.8 mAh cm^–2^ (77.7% retention) after 280 cycles. Replacing Li-In with an Si anode, which offers superior Li⁺ transport kinetics and dendrite suppression, further enhanced the cycle performance of ASSLBs [41]. The Si|LPSCl_1.5_|LPSClF_0.5_|LCO ASSLBs, cycled at 0.5 C (1.85 mA cm^–2^) with a high mass loading LCO of 25.5 mg cm^–2^, maintained 3.3 mAh cm^–2^ (129 mAh g^–1^, 92% of LCO theoretical capacity) after 280 cycles, with 86.8% retention (Fig. 3e). Remarkably, the Si|LPSCl1.5|LPSClF0.5|LCO ASSLBs, cycled at ultra-high mass loading LCO of 101.9 and 203.8 mg cm^–2^, showed ultra-high areal capacities of 15.0 and 25.7 mAh cm^–2^, respectively (Fig. 3f–h). These values correspond to high specific capacity utilizations of LCO at 147 and 126 mAh g^–1^, respectively. These results confirm the robust electrochemical compatibility between LPSClF_0.5_ SSE and uncoated LCO.

**Figure 4.**
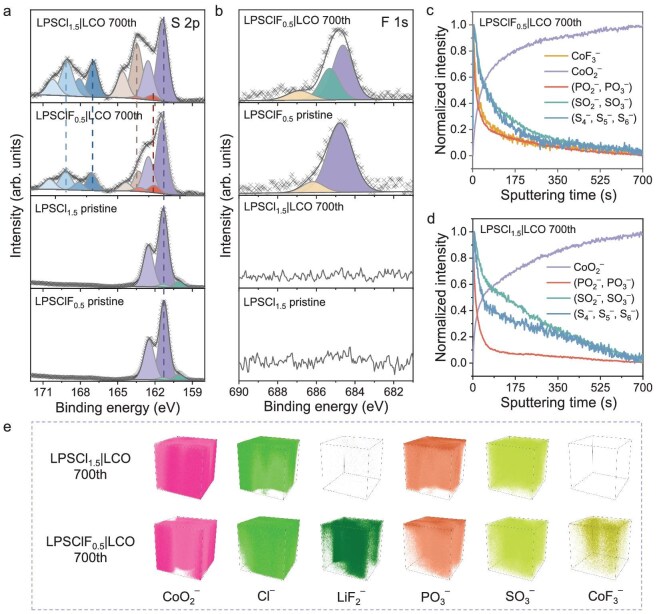
XPS evaluation and ToF-SIMS depth profiling on SSE|LCO interface for Li-In|LPSCl_1.5_|LPSClF_0.5_|LCO and Li-In|LPSCl_1.5_|LCO ASSLBs after 700 cycles. (a and b) S 2p (a) and F 1 s (b) XPS spectra. (c and d) ToF-SIMS depth profiles of LPSClF_0.5_|LCO (c) and LPSCl_1.5_|LCO (d) interface after 700 cycles. (e) The corresponding 3D view images of CoO_2_^−^, Cl^−^, LiF_2_^−^, PO_3_^−^, SO_3_^−^ and CoF_3_^−^ of LPSCl_1.5_|LCO (top) and LPSClF_0.5_|LCO (bottom).

To elucidate the SSE|CAM interfacial stabilization mechanism, the multi-modal analytical techniques, including XPS, ToF-SIMS, SEM, spherical aberration-corrected transmission electron microscopy (AC-TEM) and *in situ* EIS, were employed. XPS was conducted to quantitatively identify decomposition products. To characterize the impact of fluorine incorporation on the decomposition process, the bare LCO composite cathode based on LPSCl_1.5_ or LPSClF_0.5_ was analyzed before (0 cycles) and after 700 cycles. For comparison, XPS was also conducted on LPSCl_1.5_ and LPSClF_0.5_ SSEs (Fig. 4a and b). In the S 2p (Fig. 4a) and P 2p (Fig. S11) spectra of pristine LPSClF_0.5_ and LPSCl_1.5_, the main component with the S 2p_3/2_ peak at 161.5 eV and the P 2p_3/2_ peak at 131.8 eV corresponds to the PS_4_^3–^ groups [42], whereas the doublet at 160.1 eV can be attributed to the LiS_2_ species. After 700 cycles, the S 2p spectrum of bare LCO composite cathode based on LPSCl_1.5_ or LPSClF_0.5_ exhibits new doublets with the 2p_3/2_ peaks at 162.0, 163.5, 167.1 and 169.1 eV, suggesting the decomposition products of bridging sulfur in Li_2_S_x_, P_2_S_x_, SO_3_^2−^and SO_4_^2−^, respectively [42–44]. The sulfate content (SO_3_^2−^ and SO_4_^2−^) at the LPSClF_0.5_|LCO interface was significantly lower than that at the LPSCl_1.5_|LCO interface (Fig. S12), confirming the efficacy of fluorine in mitigating the degradation of SSE. The evolution trends of the P 2p are similar to S 2p spectra (Fig. S11). In the F 1 s spectrum, the peak at 685.3 eV corresponds to the F-Co bond, suggesting the presence of fluorine-containing CEI at the LPSClF0.5|LCO interface (Fig. 4b) [45]. These results demonstrate that the addition of fluorine effectively suppresses the formation of these resistive decomposition products and enhances the oxidation resistance and interfacial compatibility of the LPSClF_0.5_ SSE, thereby achieving superior performance. To further confirm the decomposition process in the composite cathode, depth profiling via ToF-SIMS was conducted on the bare LCO composite cathodes based on LPSCl_1.5_ or LPSClF_0.5_ after 700 cycles (Fig. 4c–e). Analysis of the ToF-SIMS depth profiling curves for normalized intensities of these ionic fragments reveals that the intensities of (PO_2_^–^, PO_3_^–^), (SO_2_^–^, SO_3_^–^) and (S_4_^–^, S_5_^–^, S_6_^–^) species of bare LCO composite cathodes based on LPSClF_0.5_ SSE are rapidly depleted (Fig 4c), in contrast to the gradual decrease observed in composite cathodes based on LPSCl_1.5_ (Fig 4d). It is demonstrated that LPSClF_0.5_ SSE effectively suppresses continuous decomposition reactions with LCO, avoiding the formation of a thick CEI, which aligns with the XPS results. The 3D reconstruction mapping discloses the distribution of corresponding species (Fig 4e), including CoO_2_^−^, Cl^−^, LiF_2_^−^, PO_3_^−^, SO_3_^−^ and CoF_3_^−^, validating that the fluorine-containing passivation layer comprises LiF and CoF_2_.

**Figure 5.**
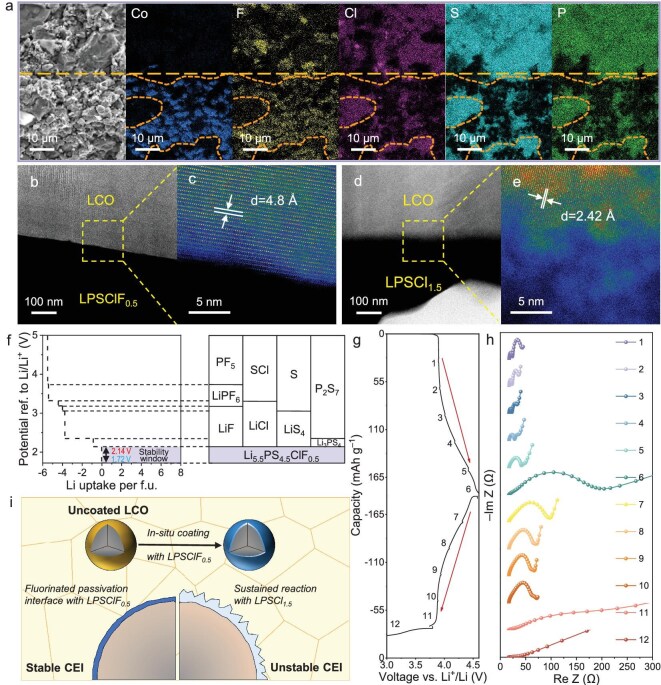
The interfacial stabilization mechanisms of SSE|LCO. (a) Cross-section SEM image and EDS images of the LPSClF_0.5_|LCO SSE interface after the 700th cycle. The yellow dashed line represents the SSE (top) and uncoated LCO composite cathode (bottom) interface. (b–e) HAADF-STEM images of LPSClF_0.5_|LCO (b and c) and LPSCl_1.5_|LCO (d and e). (f) Computed equilibrium phase behavior of LPSClF_0.5_ SSE under varying Li^+^/Li electrochemical potentials, as predicted by first-principles computational modeling. (g and h) Dynamic impedance evolution during the initial galvanostatic cycle of an LCO-based ASSLB operating within 3.0–4.6 V vs. Li^+^/Li, with synchronized voltage profiles (g) and Nyquist plots recorded during distinct charge/discharge states (h). (i) Schematic illustration of the interfacial stabilization mechanisms of SSE|LCO.

To directly observe the SSE|LCO interface condition, SEM and AC-TEM were employed, along with EDS for elemental distribution analysis (Fig. 5). Figure 5a displays a cross-section SEM image (leftmost part) and EDS mapping of the interface between the composite cathode and LPSClF_0.5_ SSE. It is revealed that fluorine is not only distributed within the SSE but also overlaps with the distribution of Co, suggesting that a portion of the fluorine has migrated to the surface of LCO, forming a fluorine-containing passivation layer. Chloride ions did not exhibit similar migration (Fig. S13). High-angle annular dark-field scanning transmission electron microscopy (HAADF-STEM) images show a distinct boundary between the LCO and the SSE (Fig. 5b and d). These samples were prepared by focused ion beam (FIB) (Fig. S14). The HAADF-STEM image confirms that the LCO in the LPSClF_0.5_ composite cathode maintains an intact lattice structure without significant damage (Fig. 5c). In contrast, the LCO in the LPSCl_1.5_ cathode exhibits an indistinct interfacial boundary and the formation of Co_3_O_4_ and amorphous phases (Fig. 5e). Bright-field images clearly present the morphology of the SSEs (Fig. S15). This corroborates that LPSClF_0.5_ SSE effectively protects the interface from persistent decomposition reactions.

First-principles computational analysis was conducted to elucidate the enhanced electrochemical stability of LPSClF_0.5_ under applied voltage gradients. The voltage-dependent thermodynamic equilibrium analysis (Fig. S16) reveals a predicted anodic decomposition potential of 2.14 V for LPSClF_0.5_. While the intrinsic oxidation potential of the LPSClF_0.5_ SSE remains relatively low, its operational anodic threshold and functional electrochemical stability window are ultimately dictated by the passivation interface formed *in situ*. Figure 5f illustrates the computed phase evolution of LPSClF_0.5_ across varying Li^+^/Li electrochemical potentials, demonstrating that fluorine incorporation plays a critical role in stabilizing the fluorinated interfacial phase. Specifically, when the SSE undergoes oxidation, it decomposes to form LiF, LiPF_6_ and P_2_F_5_. These fluorine-containing compounds exhibit a high anodic stability limit and serve as critical components of the passivating interphase layer [46]. Consequently, the electrochemical stability window of LPSClF_0.5_ can be extended. The fluorine-containing passivation layer prevents further decomposition of the LPSClF_0.5_ SSE and enables a stable interface with uncoated LCO.

EIS analysis (Fig. 5g and h) tracked impedance evolution during the first cycle (3.0–4.6 V). Nyquist plots revealed minimal total resistance of <210 Ω, along with semicircles representing charge transfer at SSE|electrode interfaces (Fig. S17). During charging (Fig. S17a–f), the resistance decreased slightly, reaching a minimum at 50% state of charge (point 3) and maximum at full charge (point 6), reflecting conductivity changes induced by LCO delithiation. During discharge (Fig. S17g–l), this trend was reversed (points 7–11), while fully discharged cells (point 12) exhibited elevated low-frequency impedance due to bulk diffusion limitations in the reduced LCO. The ASSLBs cycled at C/5 maintained stable impedance over three cycles, attributable to *in situ* formation of a protective CEI layer (Fig. S18). This stability confirms the oxidation resistance of fluorine-doped argyrodite sulfide SSE, enabling direct integration with bare LCO. The ability of fluorinated SSEs to generate stable fluorine-containing interfaces *in situ* circumvents these limitations, achieving self-stabilized SSE|CAM interfaces (Fig. 5i).

## CONCLUSION

In summary, we developed a novel class of fluorine-doped argyrodite sulfide SSEs, LPSCl_1.5–x_F_x_ (0 < x ≤ 1.5), enabling commercial LCO use for 4.6 V high-voltage ASSLBs by forming a stable fluorine-containing passivation layer on the CAM interface. Fluorine incorporation raises the oxidation limit from 2.35 to 3.5 V while maintaining high ionic conductivity of 3.3 mS cm^–1^. LPSClF_0.5_-based ASSLBs based on uncoated LCO exhibit over 700 cycles with 92.1% capacity retention at 0.2 C at 4.3 V, and stable cycling at 4.6 V when paired with lithium metal, achieving 137 mAh g^–1^ after 100 cycles at 0.5 C. Furthermore, Si|LPSCl_1.5_|LPSClF_0.5_|LCO ASSLBs with an ultra-high loading CAM of 203.8 mg cm^–2^ deliver ultra-high areal capacity of 25.7 mAh cm^–2^. Therefore, our fluorine-doped argyrodite sulfide SSEs hold significant promise for advancing ASSLBs, with their high ionic conductivity and anodic stability, driving the development of next-generation high-performance and safe ASSLBs.

## METHODS

Detailed materials and methods are available in the online Supplementary data.

## Supplementary Material

nwaf217_Supplemental_File
