# Supplementary material for: Fluorine-doped argyrodite sulfide electrolyte enables commercial LiCoO2 use for 4.6 V high-voltage all-solid-state batteries
Source: Natl Sci Rev. 2025 Jun 2;12(7):nwaf217. doi: 10.1093/nsr/nwaf217 (PMC12236154; doi:10.1093/nsr/nwaf217)
Supplement: nwaf217_Supplemental_File [file nwaf217_supplemental_file.pdf]

Supporting Information

Fluorine Doped Argyrodite Sulfide Electrolyte Enables Commercial $LiCoO_2$ toward 4.6 V High-Voltage All-Solid-State Batteries

Cong Dong,[1,4] Zhihong Bi,[2] Rui Li,[3] Yuxin Ma,[1,4] Bin Li,[1,4] Haodong Shi,[1] Zhizhen Zhang,[3] Zhong-Shuai Wu[1,5]*

[1] State Key Laboratory of Catalysis, Dalian Institute of Chemical Physics, Chinese Academy of Sciences, 457 Zhongshan Road, Dalian 116023, China

[2] China Mobile Energy Technology (Beijing) Co Ltd, Beijing, 100080, China

[3] School of Materials, Sun Yat-sen University, Guangzhou 510006, P. R. China

[4] University of Chinese Academy of Sciences, 19 A Yuquan Road, Shijingshan District, Beijing 100049, China

[5] Dalian National Laboratory for Clean Energy, Chinese Academy of Sciences, 457 Zhongshan Road, Dalian 116023, China

*__Corresponding author.__ Email: wuzs@dicp.ac.cn

Methods

Synthesis of fluorine doped Argyrodite sulfide solid-state electrolyte

A simple method based on precursor mixing and heating was used. Stoichiometric LiCl (Aladdin, 99.9%), LiF (Aladdin, 99.99%), P_2S_5, and Li_2S were mixed, pelletized, and placed in sealed quartz tubes under vacuum in the targeted proportions. The pellets were heated to 450–490 ℃ at a heating rate of 5 ℃ min^{-1} and sintered for 48 h, then slowly cooled to room temperature at 10 ℃ h^{-1}.

Materials characterization

X-ray diffraction (XRD) patterns were obtained on SmartLab with Cu k_{α} radiation in the 2θ range of 10–90 °, with samples placed on a zero-background sample holder in an Ar-filled glovebox and protected by a Kapton film. X-ray photoelectron spectroscopy (XPS) measurements were carried out on ESCALAB Xi^{+}. Cycled cathode composites of $LiCoO_2$ (LCO)-$LPSClF_{0.5}$ (cut-off 4.3 V for 700 cycles) were compared to pristine solid-state electrolyte (SSE) and uncycled cathode composite, and all measurements were conducted at the discharged state. The surface of the composite cathode was initially cleaned by Ar+ sputtering before the test to reduce the detrimental effects of surface contamination. For chemical characterization, time-of-flight secondary-ion mass spectrometry (ToF-SIMS) was performed by means of a TOF.SIMS5-100. Ar^{+} ion sputtering was also applied before the test to remove possible contamination. The measurements were conducted on the top surface of the cathode composite pellets. Material morphologies and elemental analysis studies utilized a JSM-7900F scanning electron microscope (SEM) equipped with an energy

dispersive spectrum (EDS). For atomic-scale structural characterization of the SSE|LCO composite cathode interfaces, cross-sectional specimens were first precision-fabricated via focused ion beam (FIB) milling (Helios 450HP, FEI) under cryogenic conditions, followed by atomic-resolution high-angle annular dark-field scanning transmission electron microscopy (HAADF-STEM) imaging and electron energy-loss spectroscopy (EELS) mapping using a JEM-ARM300F microscope operated at 300 kV. All specimen transfer procedures between preparation and analysis stages were executed with strictly controlled air exposure protocols (≤1 minute duration) utilizing inert atmosphere transfer modules to prevent surface degradation. All test electrodes were systematically retrieved through controlled pressure application via a mold assembly on the anode side within an Ar-atmosphere glovebox, enabling intact disassembly of the solid-state battery cell.

Electrochemical measurements

The ionic conductivity was determined through electrochemical impedance spectroscopy (EIS) measurements conducted under rigorously controlled conditions. Typically, 200 mg of SSE powder between stainless steel current collectors (10 mm diameter) via uniaxial cold-pressing at 3 tons for 60 s within an Ar-glovebox (H_2O/O_2 < 0.1 ppm), forming dense pellets for electrochemical evaluation. Impedance spectra were acquired using a Zennium Pro electrochemical workstation (Zahner) with a 10 mV AC perturbation across the frequency domain of 1 MHz to 100 mHz. For activation energy (E_a) analysis, temperature-dependent impedance measurements spanning 25–105 ℃ were performed using identical instrumentation, ensuring

isobaric conditions throughout the heating/cooling protocol.

All-solid-state lithium batteries (ASSLBs) were assembled in an ultra-dry Ar-atmosphere glovebox (H_2O/O_2 < 0.1 ppm) using $LPSClF_{0.5}$ SSE, commercial LCO cathode active material (CAM) and Li-In alloy anodes. LCO were preserved in hermetically sealed containers under Ar prior to use. First, 100 mg $LPSCl_{1.5}$ powder was initially densified under 2 tons for 60 s into 10 mm *Φ* pellets, followed by depositing 50 mg $LPSClF_{0.5}$ on one face and re-pressing under identical conditions. Composite cathodes were formulated by homogenizing LCO and $LPSClF_{0.5}$ at an optimized 80 wt% CAM to 20 wt% SSE ratio using planetary ball milling (150 rpm, 20 min). Approximately 8 mg of CAM-SSE composite (~1.2 mAh cm^{-2} areal capacity) was deposited on the $LPSClF_{0.5}$ SSE surface and consolidated under 3 tons for 180 s. For high mass loading cells, 25–200 mg cathode composite was used, and the corresponding areal capacities are labelled in the figures. On the other side of the pellet, a thin In film (10 mm diameter, 99.99%, 100 μm thickness) was attached and a Li foil (8 mm diameter, 99.99%, 50 μm thickness) was placed over the indium foil. For Si anode, a Si film (10 mm diameter, 99.5% Si + 0.5% PVDF) was attached and a Li foil (10 mm diameter, 99.99%, 50 μm thickness) was placed over the Si film. The N/P ratio is 1.3. Finally, the assembled cells were compressed under a pressure of 1 ton for 60 s, and the screws were tightened. During cycling, no additional external pressure was applied to the cell.

Galvanostatic cycling was performed on a LAND CT2001A battery system. Current densities were calculated following the conventional 1 C value for LCO (1

C = 145 mA g^{-1}). For impedance evolution studies, the ASSLB was cycled at C/5 rate with a Zennium Pro electrochemical workstation (10 mV AC amplitude, 1 MHz–100 mHz). Measurements were conducted after every 50 mins of charge-discharge.

Theoretical simulation

First-principles calculations were performed using the Vienna *Ab* Initio Simulation Package (VASP) [1]. The generalized gradient approximation (GGA) with the Perdew-Burke-Ernzerhof (PBE) functional was applied for the exchange-correlation functional [2], utilizing the projector-augmented wave (PAW) method [3]. A plane-wave cutoff energy of 520 eV was used. The electronic self-consistency loop was converged to a criterion of 10^{-5} eV, and atomic positions fully relaxed until the forces on each atom were less than 0.01 eV/Å. The pseudopotentials used in the calculations included Li_sv, P, S, Cl, and F.

The structural model of $Li_{5.5}PS_{4.5}ClF_{0.5}$ for the DFT calculations was generated by evaluating a set of 1 × 1 × 1 ordered configurations using the *enumlib* package [4]. DFT calculations were performed on the 50 configurations with the lowest Ewald energies, and the configuration with the lowest total energy was identified as the representative ground-state model.

The compositional phase diagram was constructed by comparing the relative formation energies of phases within the Li-P-S-Cl-F system. Electrochemical stability windows were assessed by constructing grand potential phase diagrams of all relevant phases of the Li-P-S-Cl-F systems in equilibrium with the chemical potential of Li [5]. The chemical potential of $\mu_{Li}(\varphi)$ is given below:

$$\mu_{\mathrm{Li}}(\varphi) = \mu_{\mathrm{Li},0} - \mathrm{e}\varphi \quad (1)$$

where $\mu_{\mathrm{Li},0}$ is the chemical potential of metallic Li, e is the elementary charge, and φ is the applied potential referenced to metallic Li.

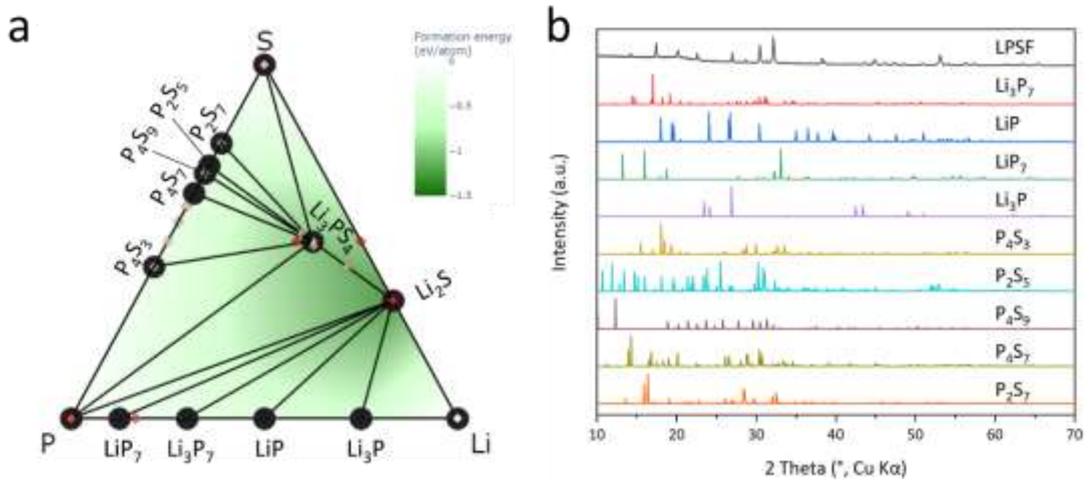

Figure S1. a) Ternary phase diagram of the Li-P-S system and b) corresponding XRD patterns of the distinct phases.

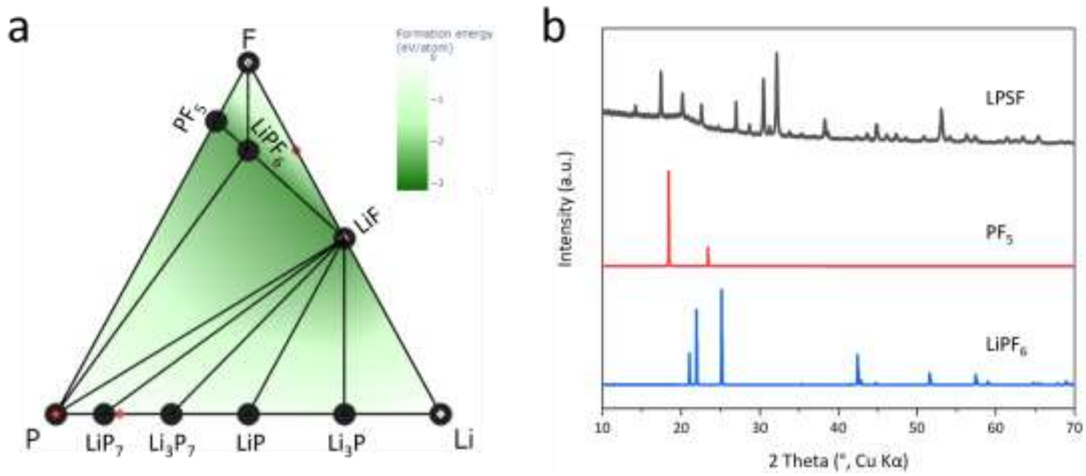

Figure S2. a) Ternary phase diagram of the Li-P-F system and b) corresponding XRD patterns of the distinct phases.

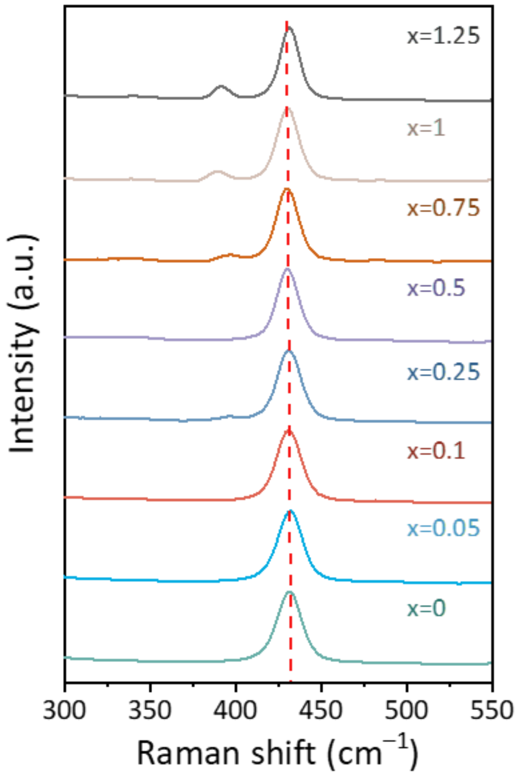

Figure S3. Raman spectra of the prepared $LPSCl_{1.5-x}F_x$ SSEs (x = 0, 0.05, 0.1, 0.25, 0.5, 0.75, 1 and 1.25).

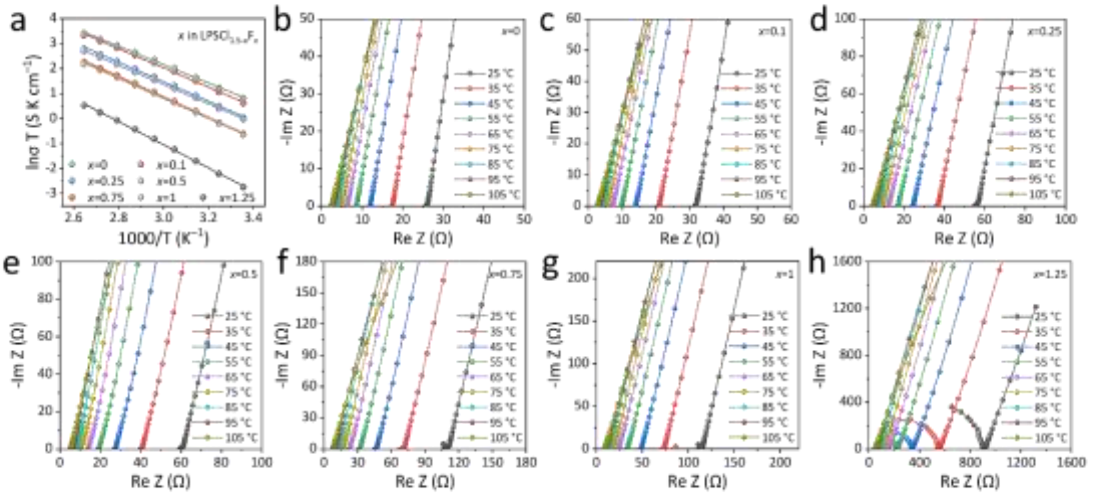

Figure S4. a) Arrhenius plots for $LPSCl_{1.5-x}F_x$. b-h) Corresponding Nyquist plots of $LPSCl_{1.5}$ (b), $LPSCl_{1.4}F_{0.1}$ (c), $LPSCl_{1.25}F_{0.25}$ (d), $LPSClF_{0.5}$ (e), $LPSCl_{0.75}F_{0.75}$ (f), $LPSCl_{0.5}F$ (g) and $LPSCl_{0.25}F_{1.25}$ (h) at each temperature used in the Arrhenius plots.

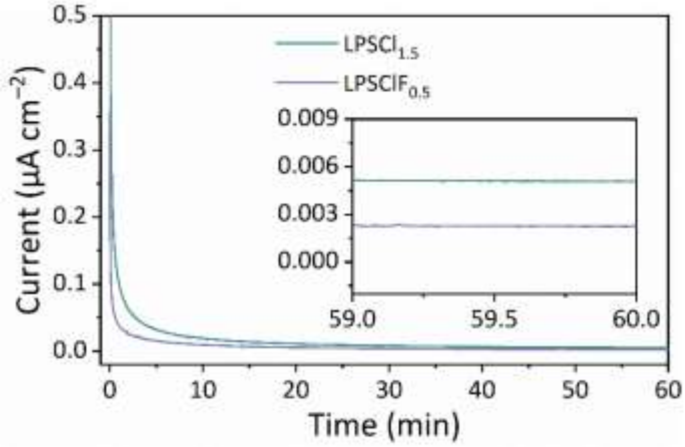

Figure S5. Comparison of electronic conductivity of $LPSClF_{0.5}$ and $LPSCl_{1.5}$ measured by the DC polarization method. Chronoamperometry result for the stainless steel|SEE|stainless steel cells with constant voltage of 1 V. The corresponding electronic conductivity of $LPSCl_{1.5}$ (green curve) is 1.56×10^{-9} S cm^{-1} and $LPSClF_{0.5}$ (purple curve) is 6.27×10^{-10} S cm^{-1}.

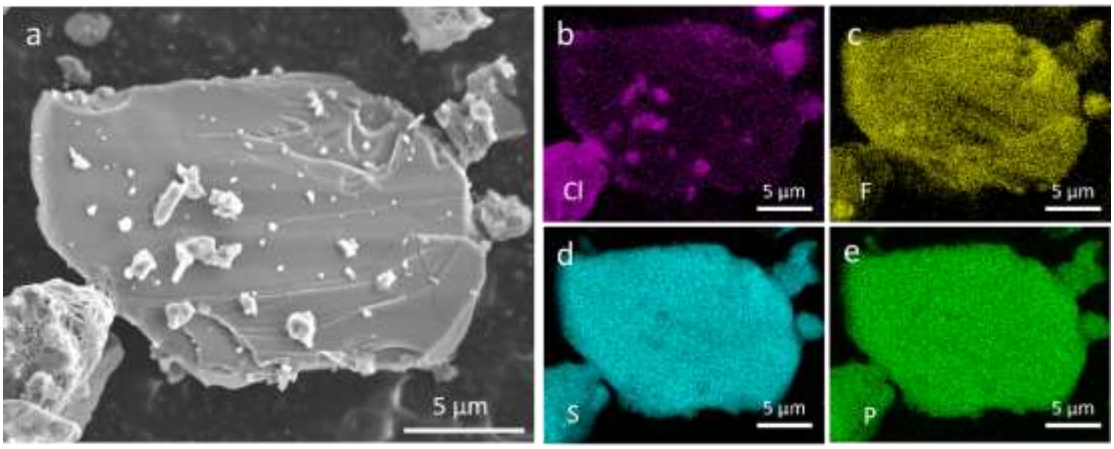

Figure S6. a) SEM image of $LPSClF_{0.5}$ SSE. b-e) Corresponding EDS elemental mapping of Cl (b), F (c), S (d) and P (e).

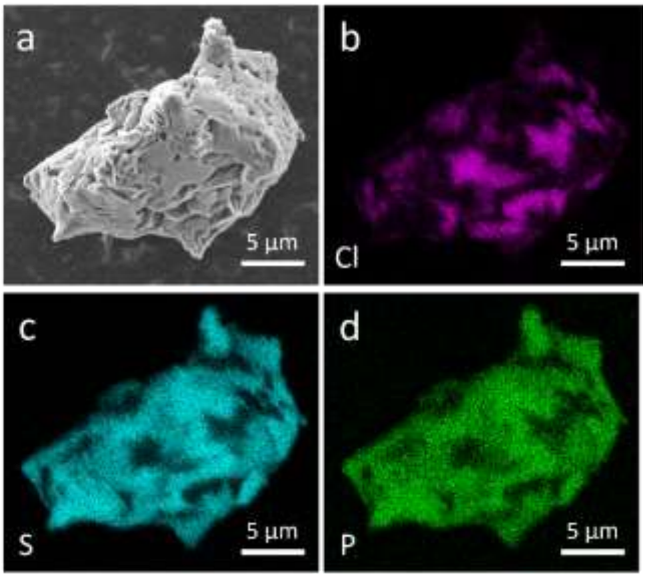

Figure S7. a) SEM image of $LPSCl_{1.5}$ SSE. b-e) Corresponding EDS elemental mapping of Cl (b), S (c) and P (d).

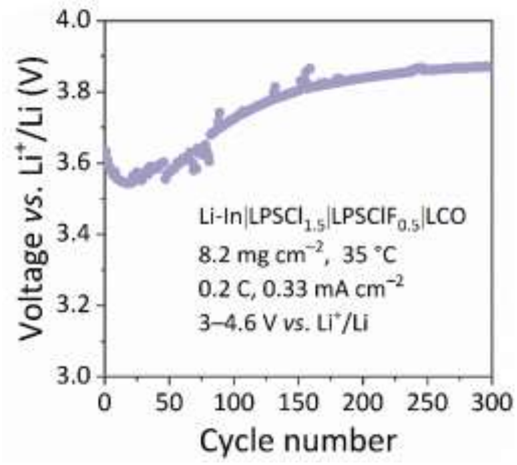

Figure S8. Discharge median voltage of InLi|$LPSCl_{1.5}$|$LPSClF_{0.5}$|LCO ASSLB at an elevated cutoff voltage of 4.6 V.

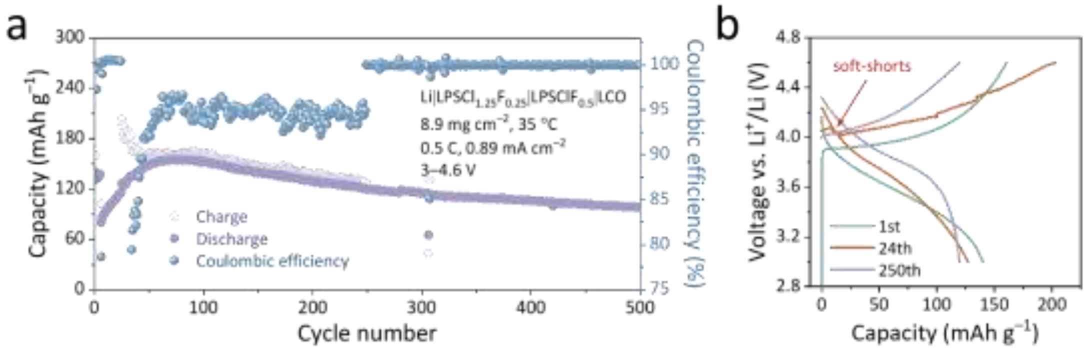

Figure S9. a) Long-term cycling of Li|$LPSCl_{1.25}F_{0.25}$|$LPSClF_{0.5}$|LCO ASSLB cycled between 3.0 and 4.6 V *vs*. Li^+/Li and b) corresponding GCD profiles.

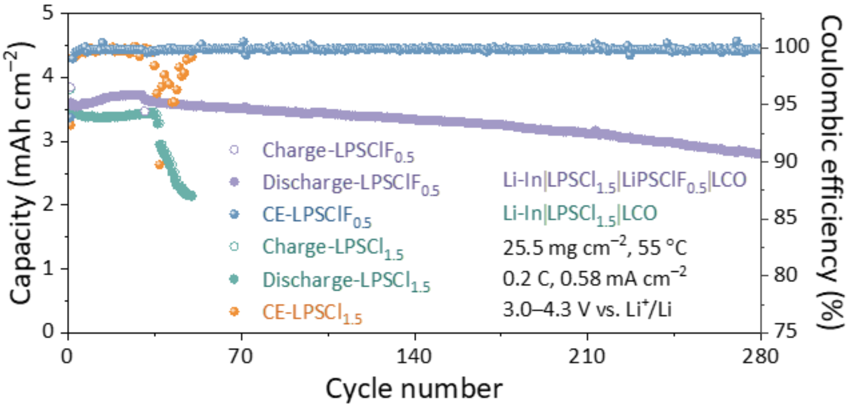

Figure S10. Cycling stability of Li-In|$LPSCl_{1.5}$|$LPSClF_{0.5}$|LCO and Li-In|$LPSCl_{1.5}$|LCO ASSLBs based on high mass loading LCO of 25.5 mg cm^{-2} under 0.58 mA cm^{-2} at 55 ℃.

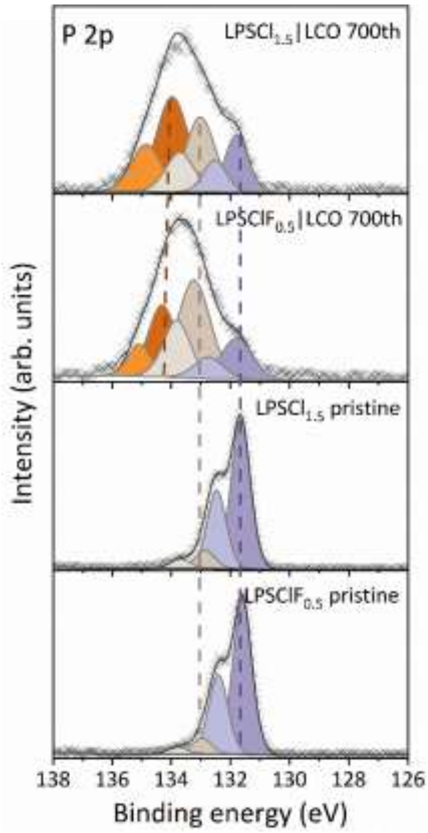

Figure S11. P 2p XPS spectra on SSE|LCO interface for Li-In|LPSCl$_{1.5}$|LPSClF$_{0.5}$|LCO and Li-In|LPSCl$_{1.5}$|LCO ASSLBs after 700 cycles, compared with the pristine electrolytes.

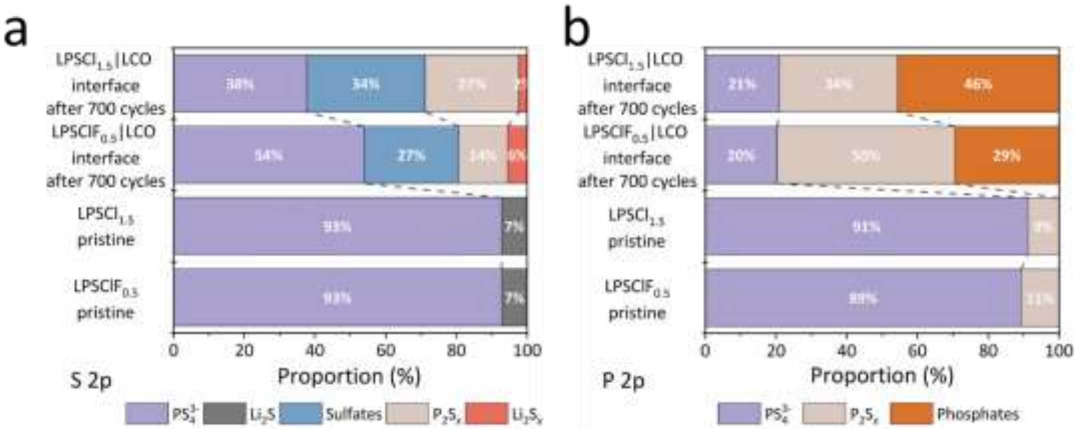

Figure S12. a,b) Semi-quantitative analyses of the S 2p (a) and P 2p (a) XPS spectra.

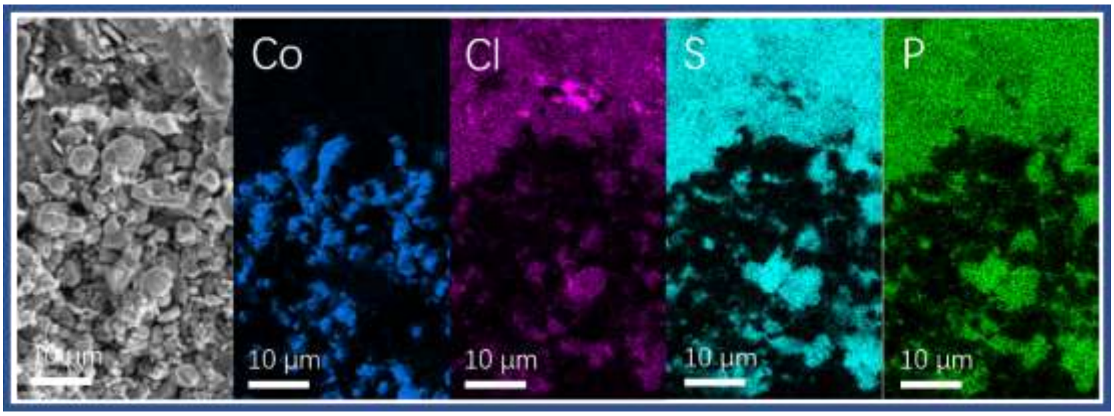

Figure S13. Cross-section SEM image and EDS images of the LCO|$LPSCl_{1.5}$ interface after the 700th cycle.

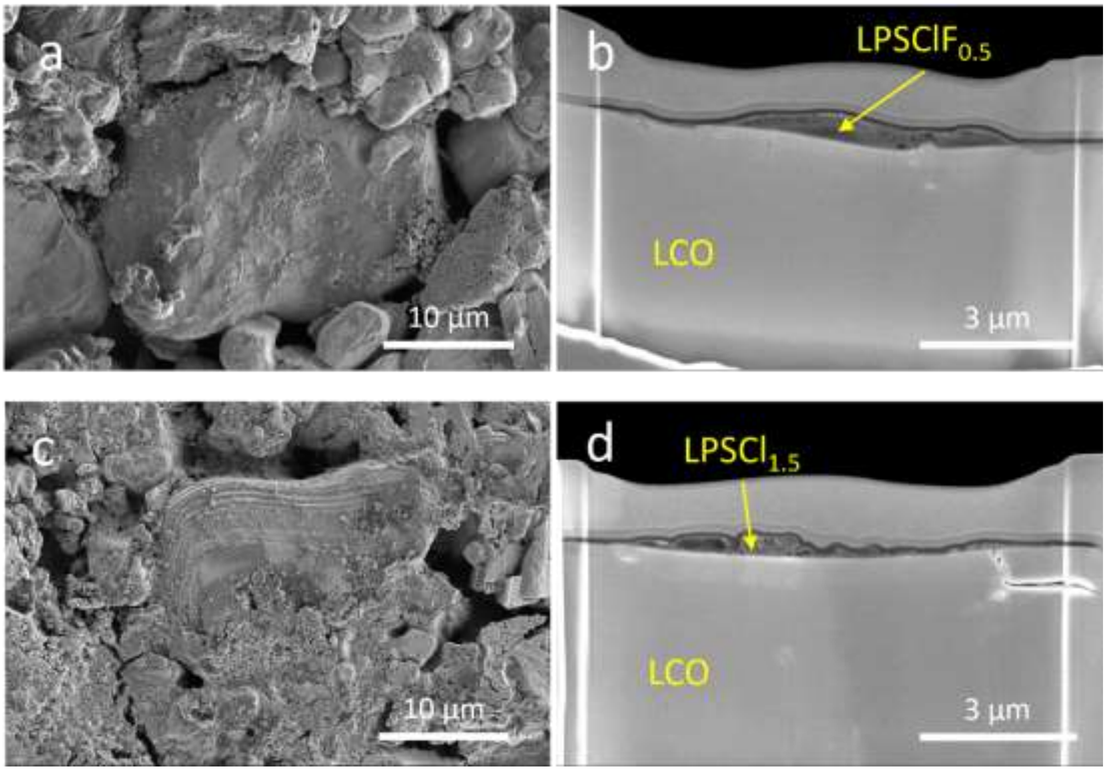

Figure S14. a,b) SEM images of LCO|$LPSClF_{0.5}$ SSE (a) and cross-section SEM images sliced by a FIB of LCO|$LPSClF_{0.5}$ SSE (b) composite cathode. c,d) SEM images of LCO|$LPSCl_{1.5}$ SSE (c) and cross-section SEM images sliced by a FIB of LCO|$LPSCl_{1.5}$ SSE (d) composite cathode.

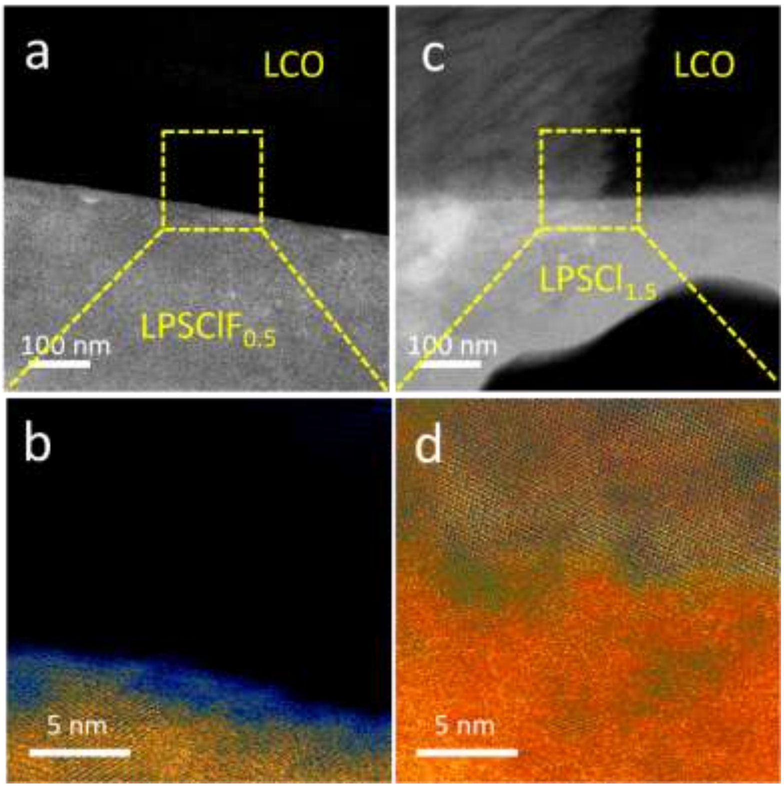

Figure S15. Bright-field images of $LPSClF_{0.5}$|LCO (a and b) and $LPSCl_{1.5}$|LCO (c and d).

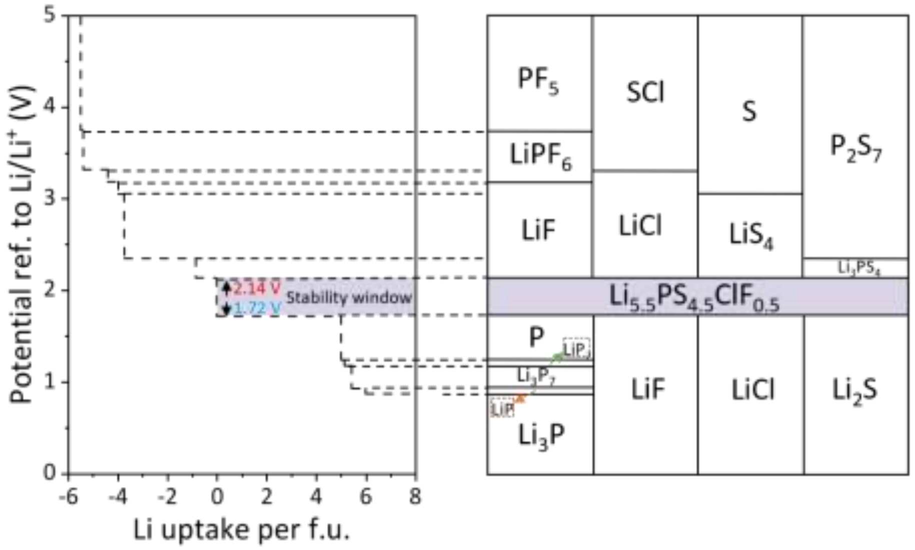

Figure S16. Phase equilibria of $LPSClF_{0.5}$ SSE at different Li/Li^+ potentials based on the first principles computation.

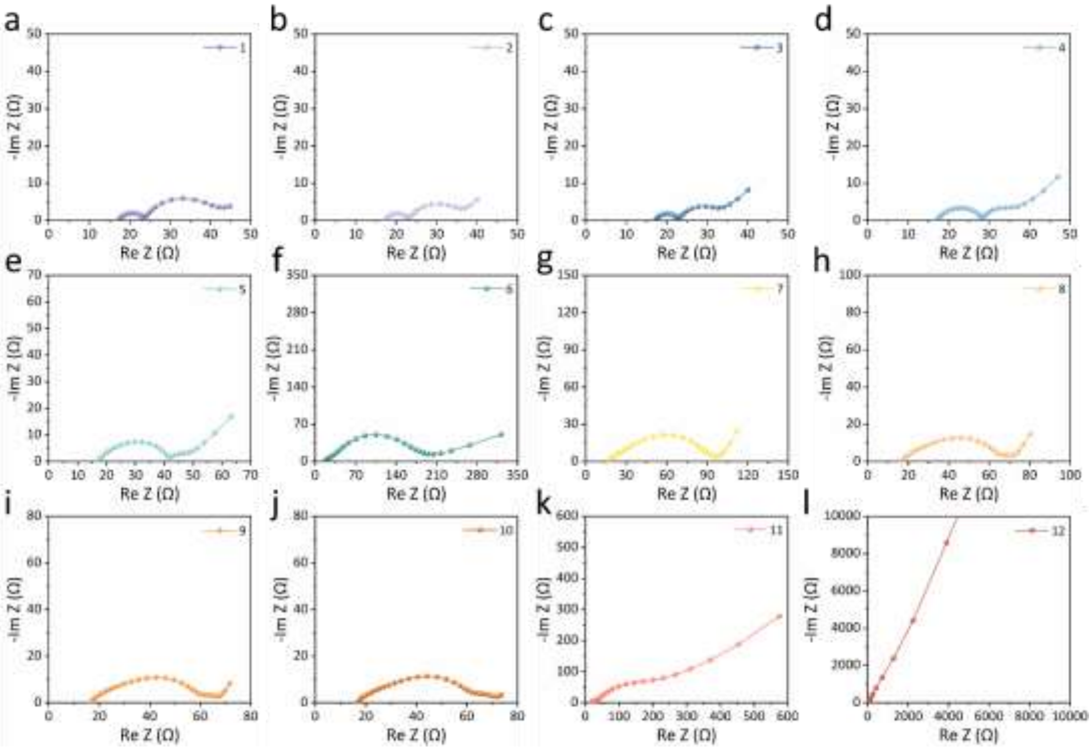

Figure S17. a-l) Nyquist plots recorded during distinct charge/discharge states of each point.

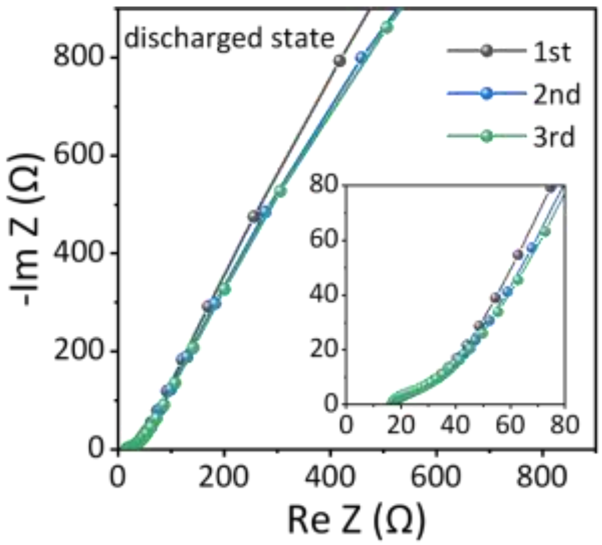

Figure S18. Nyquist plots of a LCO ASSB cycled between 2.8 and 4.6 V *vs*. Li^+/Li for 3 cycles at full discharge. The cells were equilibrated for 1 h before each test.

Table S1. Performance comparison of our ASSLSBs with other recent works.

**Solid-state electrolyte**	**Ionic conductivity ($mS\ cm^{-1}$)**	**Oxidation limit (V *vs.* Li^+/Li)**	**Active material and Areal loading ($mg\ cm^{-2}$)**	**Anode**	**Areal capacity ($mAh\ cm^{-2}$)**	**Discharge capacity ($mAh\ g^{-1}$)/Current density ($mA\ cm^{-2}$)**	**Capacity retention (%)/Cycle number**	**Ref.**
$\mathbf{Li_{5.5}PS_{4.5}ClF_{0.5}}$	3.3	3.5	bare-LCO 8.2	Li-In	1.15	140 (0.24, 35℃)	92.1 (700)	This work
			bare-LCO 25.5	Si	3.3	129 (1.85, 55℃)	86.8 (280)	This work
			bare-LCO 101.9	Si	15.0	147 (1.78, 55℃)	89.8 (20)	This work
$\mathbf{Li_6PS_5Cl_{0.3}F_{0.7}}$	0.7	/	LCO@LNO 8.92	Li	1.0	115 (0.13, RT)	95 (50)	[6]
$\mathbf{Li_{9.98}Ge_{0.99}Sn_{0.01}P_2S_{11.98}F_{0.02}}$	6.28	/	LCO /	Li	/	102.9 (0.13, 25℃)	80.1 (600)	[7]
$\mathbf{Li_6PS_5F_{0.75}I_{0.25}}$	0.15	/	$Li_4Ti_5O_{12}$ 1.0-1.2	Li	0.14	140 (0.02, RT)	75 (200)	[8]
$\mathbf{Li_2ZrCl_{6-x}F_x}$	0.321	4.25	bare-LCO 4.46	Li-In	0.39	86.4 (0.31, RT)	76 (70)	[9]
$\mathbf{Li_2ZrF_5Cl_1}$	5.5×10^{-4}	5	/	/	/	/	/	[10]
$\mathbf{Li_3InCl_{4.8}F_{1.2}}$		over 6 V	bare-LCO 7.1	Li-In	1.0	140 (0.125, RT)	72.8 (70)	[11]

LCO: $LiCoO_2$; LNO: $LiNbO_x$

REFERENCES

1. Kresse G, Furthmüller J. Efficient iterative schemes for ab initio total-energy calculations using a plane-wave basis set. *Phys Rev B* 1996; **54**: 11169-86.
2. Perdew JP, Ernzerhof M, Burke K. Rationale for mixing exact exchange with density functional approximations. *J Chem Phys* 1996; **105**: 9982-5.
3. Blöchl PE. Projector augmented-wave method. *Phys Rev B* 1994; **50**: 17953-79.
4. Ong SP, Richards WD, Jain A *et al.* Python materials genomics (pymatgen): a robust, open-source python library for materials analysis. *Comput Mater Sci* 2013; **68**: 314-9.
5. Zhu Y, He X, Mo Y. Origin of outstanding stability in the lithium solid electrolyte materials: insights from thermodynamic analyses based on first-principles calculations. *ACS Appl Mater Interfaces* 2015; **7**: 23685-93.
6. Zhao FP, Sun Q, Yu C *et al.* Ultrastable anode interface achieved by fluorinating electrolytes for all-solid-state Li metal batteries. *ACS Energy Lett* 2020; **5**: 1035-43.
7. Zhang N, He Q, Zhang L *et al.* Homogeneous fluorine doping toward highly conductive and stable $Li_{10}GeP_2S_{12}$ solid electrolyte for all-solid-state lithium batteries. *Adv Mater* 2024; **36**: 2408903.
8. Arnold W, Shreyas V, Akter S *et al.* Highly conductive iodine and fluorine dual-doped argyrodite solid electrolyte for lithium metal batteries. *J Phys Chem C* 2023; **127**: 11801-9.
9. Tang W, Xia W, Hussain F *et al.* A dual-halogen electrolyte for protective-layer-free all-solid-state lithium batteries. *J Power Sources* 2023; **568**: 232992.
10. Umeshbabu E, Maddukuri S, Hu Y *et al.* Influence of chloride ion substitution on lithium-ion conductivity and electrochemical stability in a dual-halogen solid-state electrolyte. *ACS Appl Mater Interfaces* 2022; **14**: 25448-56.

11. Zhang SM, Zhao FP, Wang S *et al.* Advanced high-voltage all-solid-state Li-ion batteries enabled by a dual-halogen solid electrolyte. *Adv Energy Mater* 2021; **11**: 2100836.
